# Learning From Multiple Representations: Prior Knowledge Moderates the Beneficial Effects of Signals and Abstract Graphics

**DOI:** 10.3389/fpsyg.2020.601125

**Published:** 2020-12-16

**Authors:** Andrea Vogt, Melina Klepsch, Ingmar Baetge, Tina Seufert

**Affiliations:** ^1^Abt. Lehr–Lernforschung, Universität Ulm, Ulm, Germany; ^2^Schweizerisches Institut für Informationswissenschaft (SII), Fachhochschule Graubünden, Chur, Switzerland

**Keywords:** instructional design, multiple representations, multimedia research, multimedia effect, signaling principle, prior knowledge

## Abstract

Multimedia learning research addresses the question of how to design instructional material effectively. Signaling and adding graphics are typical instructional means that might support constructing a mental model, particularly when learning abstract content from multiple representations. Although signals can help to select relevant aspects of the learning content, additional graphics could help to visualize mentally the subject matter. Learners’ prior knowledge is an important factor for the effectiveness of both types of support: signals and added graphics. Therefore, we conducted an experimental study situated in a university course of computer science with *N* = 124 participants. In our 2 × 2 factorial design, we investigated the effects of signals and illustrating graphics on learning outcomes and their potential interplay. Based on our regression analysis, we revealed prior knowledge as a significant moderator. Although learners with low levels of prior knowledge can profit from all types of help but still gain rather weak learning outcomes, learners with medium levels of prior knowledge profit from the synergy of both helps. With higher levels of prior knowledge, signals were particularly hampering. To improve the understanding of these supportive or hampering effects, a more fine-grained analysis of these processes and motivational effects is necessary.

## Introduction

In natural science and STEM education, learners are often confronted with highly abstract subject matters such as physical principles, mathematical systems, or computer programs. Moreover, the abstract content is often presented in abstract formats, such as mathematical or chemical formulas or computer code. Such unfamiliar, intangible formats are especially challenging for novice learners (van Meter et al., 2020). From an instructional design perspective, the question is how information on abstract subjects can be best conveyed. Very often, teachers or text-book designers use additional representations such as explanative or exemplifying texts, diagrams, or pictures to overcome these difficulties and make the abstract content easier to understand. Many findings have outlined the beneficial effects of using such multiple representations to support learning in different fields of application (van Meter et al., 2020). However, besides the well-intentioned use of additional, that is, multiple representations to enrich the abstract aspects, learners have to link the easier, accessible representations and abstract representations. Only when learners understand how the added text or picture connects to the abstract formula can they gain a deeper understanding of the abstract concept. Notably, this linking process of mentally integrating different representations of different formats is challenging for learners (Ainsworth, 2006; Seufert, 2019) and even more so for novice learners (Seufert, 2003; Ainsworth, 2014).

Because learners need to understand an abstract subject matter such as learning how to program and decipher program code, one might help them construct a mental model of the underlying system and its processes by adding a graph. As this graph is analog in nature, it can ease the construction of an analog mental model compared to the program code, which is textual in nature and thus would require translation into an analog mental counterpart (Schnotz and Bannert, 2003). However, based on the aspect that the newly added graph also poses additional requirements because it must be linked and integrated with the abstract formula, additional help could be necessary. From research on supporting learning with multiple representations, we know that signals that highlight the matching parts within multiple representations can help support the integration process (Van Gog, 2014; Richter et al., 2018). With such signals, learners can observe which elements in one representation refer to and match with which element in the other representation. Especially for analyzing the elements of an abstract formula or a piece of program code, it could be helpful to see which part of the code refers to which part of an explanatory text or illustrating graphic. As we have discussed, learners’ prior knowledge is an important influence factor for the effective use of multiple representations. However, prior knowledge not only moderates the effects of the added representations but also the effects of the additional help for integration (Seufert, 2003; Fletcher and Tobias, 2014; Van Gog, 2014; Schüler et al., 2015; Richter et al., 2018).

**FIGURE 1:**
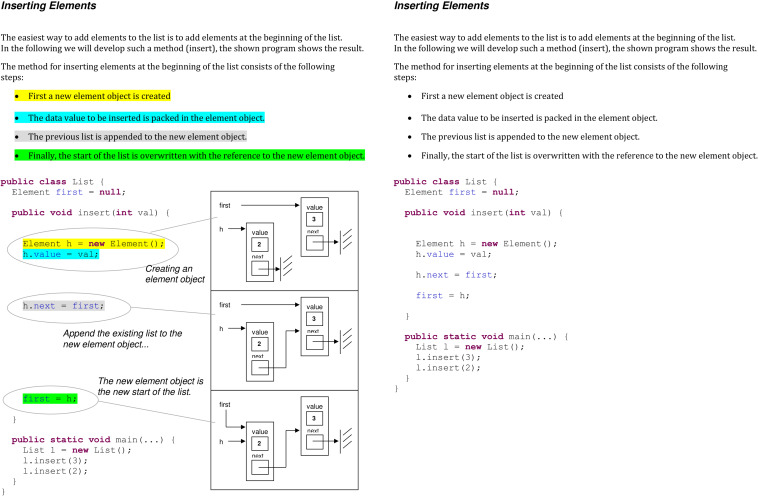
Learning material including textual and graphical representations and signaling through color coding, lines and graphic annotations on the left side and no supporting elements on the right side.

In this study, we thus examined two different instructional means to foster learning how to program. First, we included additional graphics to ease the construction of an analog mental model when managing computer code. These additional graphics were UML-charts displaying visually the architecture of the given abstract syntax. These graphical representations might support learners to build an analog mental model and therefore be reflected by an increased learning outcome. Second, we added signals to ease the mapping process when linking different representations. We implemented different signaling elements: color coding and lines to map the different representations when both text and UML-charts were given. We analyzed the effects of adding UML-charts and we explore the effects of color coding as well as adding lines on learning outcome. These signals might facilitate the mapping process of different representations and thus have beneficial effects on learning outcome. To investigate potential synergic effects, we combined both, additional UML-charts and color coding with additional lines to map the different representations (for further details see section “Learning Material” and Figure 1). Additionally, we investigated the complex interplay of learners’ prior knowledge and instructional means. To enhance the ecological validity of this research, we implemented the study into a real university course.

## Building a Coherent Mental Model by the Integration of Multiple Representations

In natural science or STEM textbooks, the learning content is often highly abstract; thus, they regularly include multiple representations, for example, text, pictures, or diagrams to illustrate or enrich the abstract content (van Meter et al., 2020). Adding pictures especially aims at fostering learners’ understanding. This more intuitive use of pictures is corroborated in the multimedia principle of Mayer (2009), which says that using text and pictures is superior to learning from text alone (Butcher, 2014). This combination activates both the verbal and the imaginary system; thus, the mentally stored information is dually coded (Paivio, 1990). Notably, pictures also ease the construction of an analog mental model. Particularly, in natural science domains, learners need to construct such a mental model that mirrors the analog structure and processes within the learners’ mind. For example, when learners want to mentally follow the steps when executing a program code, the resulting animation in their mind mirrors such a mental model. However, how is verbal information, such as in texts or formulas and pictorial information, encoded and included into a coherent mental model? The Integrated Model of Text and Picture Comprehension (IMTPC; Schnotz and Bannert, 2003) addresses this question. Two branches, one for processing text or formulas (symbolic representations) and one for pictures (analog representations), are described. These two branches describe generating an internal representation via sub-semantical (syntactical) and semantical processing of the provided external representations of the learning material. These processes are distinct in either symbolic or analog information and not only the externally presented representations are symbolic or analog in nature but also the resulting mental representations in the two branches. The model assumes that the construction of a coherent mental model, which includes information from both branches, is the main goal of learning and marks the point of deep understanding of the subject matter. As the pictorial representation already comprises an analog structure, it can be directly used as a frame for the mental model (Schnotz and Bannert, 2003). For learning with text by using the symbolic branch, an additional translation process is required: Learners need to translate the internal representation to include this information into the mental model. This last step of translating propositional, that is, symbolic information from texts into an analog mental model, is demanding (Seufert, 2019).

When learning from abstract representations such as mathematical or chemical formulas or computer code, there is an additional requirement. The language used in these formulas is not natural and thus not understandable *per se*. It first needs to be translated into natural language (Robins et al., 2003). Only then it can be translated into an analog representation. In such cases, it is plausibly especially helpful to provide analog representations such as pictures to ease at least one step of the translation process. Studies have found evidence for improved learning outcomes when adding pictures to abstract descriptive representations, even if those pictures were not always intuitive and familiar in their format (Ott et al., 2018; Vogel et al., 2019; Malone et al., 2020).

Overall, to aid to construct a mental model and thus foster comprehension and transfer, pictures can be used as a general concept to frame the mental model, and textual details can be added to refine it (Mayer, 2009; Schüler et al., 2015).

Although learning can be fostered and simplified by using pictures in addition to text, building a coherent mental model, remains challenging (Gentner, 1983). Learners are required to create referential connections between corresponding elements and structures in the different representations (Seufert, 2003). To support the learners while mapping the different representations, supportive elements might be included in the learning material.

The signaling principle (Mayer, 2014; Van Gog, 2014) indicates that signals, for example, through color-coding, highlights, or adding lines, help with mapping over various representations. Hence, signals aid cognitive processes by making the relevant and linkable information more salient on a surface feature level (Seufert and Brünken, 2006).

The two support systems, added graphics and added signals, can have synergetic effects. The mapping help of signals is especially necessary and relevant when pictures are added. However, even without the signals, the graphics might be helpful, and even without the graphics, the signals might help select what is relevant and how other representations, such as text and formulas, can be mapped and integrated. Despite the interplay between the two supportive means, an additional factor interacts with the two of them, namely, learners’ aptitudes. Learners’ prior knowledge affects whether the graphic or signaling supports the learner to process information more deeply because it determines the available resources for such a deep-level approach (Richter et al., 2018).

### Influence of Learner’s Aptitudes

Whether learners benefit from the presence of instructional support is highly dependent on their prior knowledge (Richter et al., 2016; Seufert, 2019). Based on the idea of the ability-as-compensator hypothesis of Mayer and Sims (1994), instructional support is more important for learners with low prior knowledge. Learners with high prior knowledge do not depend on this support. They compensate for the given requirements by using their pre-existing mental representation (Richter et al., 2016). Thus, they might even suffer from additional support that could hinder their usual strategies. Such detrimental effects have been shown in many studies referring to the expertise reversal effect (Kalyuga et al., 2003; Kalyuga, 2009). The moderating effects of learners’ prior knowledge have been refined by Seufert (2003) who further differentiates between learners with a very low level of prior knowledge and learners with medium levels of prior knowledge. Only with at least some prior knowledge are learners able to understand the learning material and use the given support. For example, if signals highlight relevant words or pictorial entities and learners do not know what these words or picture elements mean, they will be unable to map anything, at least not on a semantic level with deeper understanding. Learners with a medium level of prior knowledge are able to use the help, are still in need of it, and thus would profit the most.

The following question now arises: How do the two supportive means, that is, adding graphics to symbolic representations and adding signals, interact with learners’ prior knowledge? Using graphics is particularly helpful for learners with lower but still sufficient prior knowledge (Fletcher and Tobias, 2014; Schüler et al., 2015). They can use the pictorial information to build a general mental model, which can be enriched with further details from the text (Schüler et al., 2015). Because learners with low prior knowledge are more dependent on bottom-up processes to build their mental representations and integrate them into a mental model, pictures can be helpful for organizing the details into one coherent model (Schüler et al., 2015). However, even if learners manage to construct a mental model, this model must not be coherent such that it includes well-integrated information from the different sources. To assist this process of connecting and mapping the suitable elements, signals come into play (Scheiter and Eitel, 2015). Again, learners with low prior knowledge should benefit from surface-level supportive elements, such as signaling through color-coding, because they are challenged to find the relevant information in the given learning material (Richter et al., 2018). As aforementioned, their prior knowledge must be sufficient to understand the meaning of the highlighted elements. Learners with expertise can easily detect the relevant words or elements in a formula or picture. Thus, they do not need additional highlights, and by contrast, experience the signals as a distraction that needs to be ignored and thus might impair performance.

### Present Study

This study aimed at investigating, under the consideration of learners’ prior knowledge, the effects of additional graphics and signals for learning how to program. We chose this subject matter because it is prototypically abstract and also prototypical for STEM education. From a cognitive perspective, the learning process can be considered similar to learning a foreign language because conventions first need to be learned and understood in a complex learning process (Robins et al., 2003). In typical computer science learning environments, two textual representations of the learning content are included: explanatory text and computer code. These two representations of the learning content might emphasize different aspects. The explanatory text includes explicit process information and describes what should occur. The code includes functional information about the programming language, such as how variables are implemented and handled (Kunkle and Allen, 2016). On a syntactic level, learners read textual code snippets, take in the different words, and can repeat them (Schnotz and Bannert, 2003). In the next step, they have to extract the semantics, that is, the propositional meaning of the code. This includes understanding the meaning of parts of the code and their inter-relational structure and enables learners to construct a propositional representation of the network of information (Schnotz and Bannert, 2003). When learners understand the meaning of the code, they can construct an analog mental model. This mental model contains all the relevant structures and processes the code described (Robins et al., 2003). Using graphics might provide information on hierarchical or spatial structures and temporal aspects of the syntax which are not obvious in the symbolic representations (Vogel et al., 2019). Computer science education often uses graphics displaying the architecture and the implementation of the respective code called Unified Modeling Language or short UML-charts.

Particularly for novices adding graphics, such as UML-charts, this should facilitate building a mental model (van Meter et al., 2020). However, adding graphics adds the challenge of mapping the corresponding information of different representations (Gentner, 1983). Adding signals by using color-coding, linking lines, or adding annotations might help to map and integrate the corresponding information of these multiple representations. Because mapping is challenging for novice learners, they should particularly benefit from signals (Richter et al., 2018) because their prior knowledge is still sufficient to understand the highlighted entities (Seufert, 2003). Hence, the combination of two different supportive elements (added graphics and signaling) seems to be very promising and might have a synergetic effect on the learning outcomes of novice learners (Kalyuga et al., 2003; Kalyuga, 2009; Magner et al., 2014; Clinton et al., 2017).

As learners with more prior knowledge already have an existing mental model, the effects of adding graphics and signals might change (Kalyuga et al., 2003; Schnotz and Bannert, 2003; Kalyuga, 2009; Schüler et al., 2015). Findings have implied that the interaction of different supportive elements in combination with prior knowledge might reveal a non-linear relationship (Seufert, 2003). Learners with the lowest levels of prior knowledge might not be able to profit from help, learners with still low but sufficient levels will benefit, and learners with high levels of prior knowledge will no longer need the help and might become distracted. Overall, prior knowledge seems to be a potential moderator whether or not learners benefit from adding graphics or signals. Based on these assumptions, we propose hypotheses 1 (H1) and 2 (H2):

H1. The two instructional means (graphics, signals) have a synergetic effect on learning outcome, which is reflected by a significantly higher learning outcome in the experimental condition where both instructional means are combined, followed by each mean condition alone. The lowest learning outcome is expected when learning with the text-only condition.

H2. Moreover, we expect that prior knowledge significantly moderates the relationship between the help condition and learning outcome (Seufert, 2003).

## Materials and Methods

This study was integrated into the regular curriculum of computer science students at a German university. The lecture “General Computer Science” was accompanied by an exercise course. The study was integrated into this exercise course and lasted 2 weeks.

### Study Design and Participants

A 2 × 2 between-subject design with learning material about classes and objects, and linear lists, both important for Java programming, was realized. Independent variables were additional graphics (with or without) and signals (with or without). Differences between groups should be reflected in the learning outcome as the dependent variable. As control variables, age, sex, prior knowledge, and verbal and spatial abilities were assessed.

Some participants did not attend one of the sessions; thus, the initial sample of 135 decreased to *N* = 124 participants, and we included the latter in the analysis. Participants’ mean age was *M* = 21.40 (*SD* = 2.00), and 62 of them where male. The participants were randomly assigned to one of four groups: no signals, text-only (*n* = 33); no signals, text and graphics (*n* = 29); signals, text-only (*n* = 31); and signals, text and graphics (*n* = 31; Table 1).

**TABLE 1:** Distribution of sexes and age overall and in each experimental condition.

Experimental condition	Male (%)	Age *M* (*SD*)
Overall (*N* = 124)	62.10	21.40 (2.00)
No signals, text-only (*n* = 33)	69.70	21.94 (2.08)
No signals, text & graphic (*n* = 29)	62.07	21.14 (1.73)
Signals, text-only (*n* = 31)	51.63	20.87 (1.80)
Signals, text and graphic (*n* = 31)	64.52	21.61 (2.23)

### Material

In the paragraphs in this section, the learning material and tests used are described in more depth.

#### Learning Material

The learning material was a booklet with a maximum of 18 pages. Depending on the experimental group, the learning material contained additional information on the supporting elements (graphics or signals). There were four versions of the learning material: (1) plain text, (2) text with graphics, (3) text with signals, and (4) text with graphics and signals. Concerning the first factor, adding graphics, the learning material included either only textual representations (explanatory texts and computer codes) or textual and graphical representations (explanatory texts, computer codes, and graphical representations in form of UML-diagrams). The second factor concerned signals and the learning material either was without signals or contained signals through color-coding to map corresponding concepts in the different representations in the learning material. In the text with signals condition, corresponding elements in the explanatory text and the computer code were marked in the same color. If both instructional means (graphics and signals) were combined, signaling was additionally realized by adding lines to indicate which parts of the UML-diagrams correspond to specific computer code parts. Moreover, in this condition, the italic text was included (graphic annotations) to signal which parts of the graphic corresponded to the text. Figure 1 shows an example of one page for the group with textual and graphical representations and signals and on the right side the material without supportive elements.

#### Prior Knowledge Test

To measure prior knowledge different approaches exist. Using multiple-choice, open, closed, or mapping questions to measure domain-specific prior knowledge was described to be an externally valid measurement method (Dochy et al., 1999). Many prior studies used questionnaires including these question types to measure domain-specific prior knowledge (e.g., Richter et al., 2018; Seufert, 2019). The prior knowledge test in this study comprised five tasks for the topic classes and objects (part 1) and five tasks for the topic linear lists (part 2), which were constructed by field experts as relevant and valid tasks for the given content. The test comprised three multiple-choice questions, three mapping questions, one cloze question, one task to locate errors, and two open questions. The total points possible on the prior knowledge test was 22 in part 1 and 21 in part 2.

#### Posttest

The knowledge test after learning comprised six tasks for the topic classes and objects (part 1) and six tasks for the topic linear lists (part 2). Total points possible was 34 points in part 1 and 38 points in part 2 of the knowledge test. The knowledge test included two closed and one mapping question, six multiple-choice questions, and three open questions to write programming code.

#### Subjective Ratings of the Learning Material

Learners rated the learning material with regard to its comprehensiveness, helpfulness, and effort necessary on a five-point Likert scale. The items were formulated to refer to the different supportive elements in their experimental group. Learners rated the elements of the learning material that they received in their experimental condition, namely, graphics or signaling (color-coding, lines, graphic annotations). For instance, the subjective rating for color coding was, for comprehensiveness*: While working through the learning material the colored highlights contributed to a better understanding*; for helpfulness: *While working through the learning material the colored highlights were helpful*; for effort: *While working through the learning material the colored highlights were hard to understand.*

#### Learners’ Aptitudes

To assess the control variables, we used different standardized tests. Spatial abilities abilities were measured with the card rotation and the paper-folding subtests of the Kit Of Factor-Referenced Tests (Ekstrom et al., 1976). The card rotation test presented 10 items. For each item, an object was given. Participants had to decide if the other eight reproductions of the given object were either rotated or flipped. The paper-folding test comprised 10 items. For each item, the steps of folding a sheet of paper were displayed, where in the final step, a hole was punched. Participants had to select one of five presented alternatives regarding how the paper would look when it was unfolded again. For each subtest, participants had 3 min. Both, paper-folding and card rotation scores, were first standardized into a percent-correct score and then integrated into one spatial ability score. Verbal abilities were assessed on three subscales of the KFT 5-12 + R (Heller and Perleth, 2000). The used subtests were short versions of the three verbal tests (V-Test 1, V-Test 2, and V-Test 3). In V-Test 1, we used 15 items with a middle level of difficulty and learners had 3 min to answer the items. In the test, a word was given, and five others were presented. Learners had to decide which of the five words best fit the given word. In V-Test 2, learners also had 3 min for 15 items with a medium level of difficulty. In this test, three words that have something in common and a selection of five additional words were presented. The subjects were instructed to mark one of the five additional words that they thought connected best with the prior three. In V-Test 3, 10 items of medium difficulty were used, and again, the subjects had 3 min to answer all the items. In this test, except for word-pairs (e.g., big:huge), an opposite word was given (e.g., small) based on the first pair, for the second word, a fitting partner had to found out of five given words.

#### Procedure

First, learners were informed of the procedure of the study and signed an informed consent form. All participants were aware that they could withdraw their data at any point in the study without having any disadvantage. The study comprised two sessions. The first session was part of the participants’ university course, and after an introduction, each participant filled out a questionnaire that elicited demographic data and took the prior knowledge test. Afterward, participants were randomly assigned to one of the experimental groups and the learning material was distributed. Everyone received information on how to work with the learning material for the next 2 weeks. During the next 2 weeks, participants could study with the material, as they would for their normal lectures. Their only instruction was to document the amount of time spent studying the material. The second session was again situated as part of the students’ course and started with the knowledge test for the learned content. Afterward, spatial and verbal abilities of participants were assessed.

## Results

### Descriptive Results

In Table 2, learners’ prior knowledge and learning outcome were on a medium level both overall and in each group.

**TABLE 2:** Means and standard deviations of the experimental conditions.

	No signals text-only *M* (*SD*)	Signals text-only *M* (*SD*)	No signals text and graphic *M* (*SD*)	Signals text and graphic *M* (*SD*)
Prior knowledge (%)	75.12 (11.77)	72.16 (15.02)	72.16 (15.65)	73.14 (11.74)
Learning outcome (%)	52.70 (10.00)	50.16 (10.71)	50.38 (10.34)	52.90 (9.38)
Verbal ability (%)	73.48 (4.94)	73.88 (3.35)	74.30 (4.33)	71.93 (6.82)
Spatial ability (%)	74.50 (15.00)	73.50 (17.50)	71.50 (17.50)	80.50 (12.00)
Study time (hours)	1.42 (1.33)	1.17 (0.60)	1.22 (0.46)	1.19 (0.71)

To ensure that no systematic group differences existed between the experimental conditions, a MANOVA was conducted with study time, prior knowledge, and verbal and spatial ability as dependent variables. It revealed no significant differences between the groups (*F* < 1, *p* = 0.564). When performing a Shapiro–Wilk test, multivariate normal distribution can be assumed for the variables learning outcome and prior knowledge for each experimental subgroup (*p* > 0.050), and the variances can be classified as homogenous based on the Bartlett’s test (*p* > 0.240). When necessary for analysis, all continuous input variables were mean-centered before analysis, and grouping variables were dummy coded.

### Learning Outcome Depending on the Available Instructional Means

We expected (H1) a synergetic, beneficial effect of the two instructional means graphics (UML-charts) and signaling (color coding, lines). The group with graphics and signaling was expected to outperform the three other groups. For each instructional mean (signals or graphics), we expected a higher learning outcome compared to the baseline condition with no signals and no graphics. However, we found no significant differences in the groups for the overall learning outcome without considering the learners’ aptitudes (*F* < 1, n.s; Table 2). Therefore, no significant main effect was found neither for each instructional mean nor for their synergic effect on learning outcome.

### Learning Outcome Depending on Experimental Condition and Learner’s Aptitude

In H2, we predicted the moderating effect of prior knowledge in combination with the two instructional means (for further details, see Supplementary Material). Therefore, we set up different regression models (Hayes, 2015). As the variables spatial and verbal ability significantly correlated with learning outcome (*r*_*verbal*_ = 0.29, *p* < 0.001; *r*_*spatial*_ = 0.43, *p* < 0.001), these variables were included as covariates in the analysis. Prior knowledge was included as a moderator, and because the non-linear relationship between prior knowledge and learning outcome has been described, non-linear models were also included in the testing hierarchy (for further details and justification, see Supplementary Material; Hayes, 2015; Richter et al., 2018).

The best model, based on adjusted *R*^2^, was a multiple regression model including a quadratic trend for prior knowledge (Table 3). Overall, the chosen model explained 60% of the variance in learning outcome, which can be classified based on Cohen (1992) as a large effect [*F*(10,113) = 19.55, *p* < 0.001, *R*^2^*_*adj*_* = 0.60, *f^2^* = 1.22].

**TABLE 3:** Multiple regression model including prior knowledge as quadratic moderator.

Variable	Estimate	Standard error	*T*-value	*P*-value
Intercept	0.80	1.34	0.60	0.551
Prior knowledge	1.17	0.11	10.74	< 0.001***
Graphical help	–3.90	1.93	–2.02	0.046*
Signaling	–1.11	1.98	–0.56	0.576
Spatial ability	5.46	2.08	2.63	0.010*
Verbal ability	0.40	0.12	3.34	0.001**
Prior knowledge^2^	–0.04	0.03	–1.26	0.210
Graphical help*Signaling	6.41	2.98	2.15	0.034*
Prior knowledge^2^ *Graphical help	0.09	0.03	2.72	0.008**
Prior knowledge^2^ *Signaling	0.02	0.04	0.43	0.670
Prior knowledge^2^ *Graphical help*Signaling	–0.13	0.06	–2.05	0.043*

*p < 0.05, **p < 0.01, ***p < 0.001.

**FIGURE 2:**
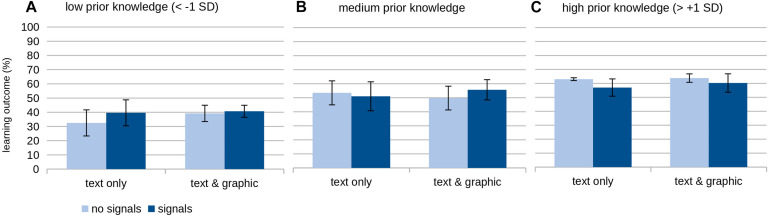
Means and standard deviations of the learning outcome depending on the different help conditions.

The interaction of graphical help and signaling was significantly moderated by learners’ prior knowledge (β = −0.13, *SE* = 0.06, *t* = −2.05, *p* = 0.043). Therefore, H2 was supported by the data: A different pattern for the effects of the two supportive elements depending on the level of prior knowledge can be described as follows (see also Figure 2).

In this study, the amount of data was insufficient to test for significant differences in the subgroups based on prior knowledge and the two supportive elements. Therefore, the second-order interaction was analyzed on a descriptive level based on three levels of prior knowledge inserted in the regression: low (below one standard deviation below the average), medium (between one standard deviation below and above the average), and high (over one standard deviation or higher over the average).

Overall, learners with low prior knowledge achieve a better learning outcome in each of the instructional support than for no additional support. They (Figure 2A) achieved the lowest learning outcome without any support from graphics or signals. Adding help by graphics or signals to the learning material improved the learning outcome, but it remained at a rather low level. Learners with medium prior knowledge (Figure 2B) show the best learning outcomes with graphical help and signaling. Upon closer examination of the learning outcome of the subjects with medium prior knowledge, we observed that the interplay of the two supportive elements was slightly more beneficial than no additional support. Compared to these two conditions, only adding graphics or signals led to a lower learning outcome. In the group of learners with high prior knowledge (Figure 2C), adding signals seemed to reduce the learning outcome because they performed better under the conditions without signals.

### Subjective Rating of Mental Effort

To evaluate learners’ compliance, we asked them for their invested effort. Overall, the scores were very low (Table 4).

**TABLE 4:** Means and standard deviations of the subjective rating on a five-point Likert scale.

	All groups *M* (*SD*)	No signals text only *M* (*SD*)	Signals text only *M* (*SD*)	No signals text + graphic *M* (*SD*)	Signals text + graphic *M* (*SD*)
**Comprehensiveness**					
Graphics	4.50 (0.65)	–	–	4.62 (0.56)	4.39 (0.72)
Color coding	4.31 (0.99)	–	4.3 (0.95)	–	4.32 (1.05)
Lines	4.13 (0.81)	–	–	–	4.13 (0.81)
Graphic Annotations	3.65 (0.91)	–	–	–	3.65 (0.91)
**Helpfulness**					
Graphics	4.48 (0.72)	–	–	4.52 (0.74)	4.45 (0.72)
Color coding	4.30 (0.88)	–	4.33 (0.99)	–	4.26 (0.77)
Lines	4.19 (0.87)	–	–	–	4.19 (0.87)
Graphic Annotations	3.68 (0.91)	–	–	–	3.68 (0.91)
**Effort**					
Graphics	1.93 (0.95)	–	–	1.72 (0.8)	2.13 (1.06)
Color coding	1.80 (0.91)	–	1.67 (0.84)	–	1.94 (0.96)
Lines	1.77 (0.99)	–	–	–	1.77 (0.99)
Graphic Annotations	2.16 (1.04)	–	–	–	2.16 (1.04)

*Depending on the experimental conditions learners only rated those supportive elements they received.*

## Discussion

Learning to program has several challenges. When using different components (e.g., explanatory texts or UML-charts) in the learning material, learners must process and integrate the information provided in the learning environment. Logical and syntactical content must be learned, understood, and integrated into one coherent mental model. Our findings indicate that the question of how to support learners by using different supportive elements in the learning material is not simple. To face the challenge of designing appropriate learning materials, learners’ prior knowledge must be considered.

### Learning Outcome Depending on the Experimental Condition

Analyzing the effects on a learning outcome without considering learners’ prior knowledge, we found neither a synergetic nor a beneficial effect when combining the two instructional means graphics and signaling. Thus, we found no multimedia effect and no positive effect of signaling, which contradicts the literature and H1. Namely, we found the highest learning outcome for the group without any supportive elements, followed by the learners who received the combination of graphics and signaling.

We now question why the two types of instructional support did not reveal the expected effects. Maybe the graphics were unsuitable or the signals did not highlight the most important aspects. Such a critical analysis could be adequate. However, the results for learners with different levels of prior knowledge reveal a positive effect of either help or their combination. Thus, the question should not be why the help works or does not work but for whom the help works or does not work. We discuss these differentiated results in the next section.

Nevertheless, one argument should be considered when explaining the overall effects. We found rather low levels of learning outcomes for approximately 50% of all groups. Even for learners with higher levels of prior knowledge, the results were only approximately 60%. This hints at a compliance problem. Even if the study was conducted as part of a facultative university course, the results did not affect the students’ grades; thus, they did not expend much effort. To ensure higher compliance, the tasks should be credited. In further research, learners’ motivation should also be assessed to analyze the effects on effort and learning outcome.

### Learning Outcome Depending on Experimental Condition and Learners’ Aptitudes

In H2, we expected a significant interaction between the two instructional means and learners’ prior knowledge. The results of our study revealed a complex interplay of prior knowledge and the different supportive elements. To provide insights into this moderating effect, we discuss the results for the three previously defined prior knowledge groups based on their descriptive pattern.

Descriptively, learners with *low prior knowledge* gained benefits from every help condition compared to the control group, who learned with text-only. Hence, the expected positive effect of adding abstract graphics for learning was supported by the present finding and is hence in line with prior findings (Fletcher and Tobias, 2014; Schüler et al., 2015). Presenting information in a pictorial manner, in our study, as UML notation, helps learners with low prior knowledge to build a mental model. General aspects such as hierarchical structures and dependencies can be learned from the graphics. Detailed information from textual or pictorial representations is inserted into the mental model afterward (Schüler et al., 2015).

Furthermore, we found a beneficial effect of signaling on learning outcomes for learners with low prior knowledge. Our results are in line with the findings of de Koning et al. (2007). They described that attention guidance through signaling can be supportive by improving the effectiveness of finding relevant information. Particularly with *low prior knowledge*, they are supported because they lack appropriate cognitive schemata (de Koning et al., 2007). Color-coding goes along with guiding learners’ attention because salient information is primarily perceived (Ozcelik et al., 2010). Shifting attention to relevant information is crucial for further integration processes. Signaling helps detect relevant information and fosters mapping on the surface level but does not guarantee a deeper understanding of the content (Seufert, 2019). The results of this study indicate that learners with *low prior knowledge* can benefit from signaling, to gain a basic level of knowledge.

Our findings also imply a synergetic effect of the two supportive elements for the group with *low prior knowledge*. Adding signals to the graphics seems to facilitate the mapping and integration process of the corresponding information. Nevertheless, independent from the type of support, these learners did not reach a high level of learning outcome overall. Thus, even with help, they only learned on a basic level and could not use help as effectively as intended. For such a deep-level approach, they would have needed more prior knowledge.

Regarding the group of learners with *medium prior knowledge*, we found effects that were more complex than initially assumed: These learners either need the combination of signaling and graphics or no additional help. Neither graphics nor signals alone revealed positive effects.

Although learners with *low prior knowledge* mostly rely on bottom-up processes, learners with *medium* or *high prior knowledge* are also able to use their existing concepts for additional top-down processes. This idea is in line with the findings of Kriz and Hegarty (2007), that is, learning for learners with medium prior knowledge went along with a complex interplay between top-down and bottom-up processes. Thus, these learners are best assisted when the learning material provides a graphic that adds to their existing knowledge and provides an analog frame for the mental model in which further information from the text and the computer code could be included. To be able to link this frame with those details from the text, code, or propositions from their knowledge base, they nevertheless need mapping help in form of signals; then, both instructional means act synergistically. With either, for the graphic alone, the frame could not have been enriched with the necessary details, and with the signals alone, the frame may still have been too fragmentary.

However, the following question remains: *Why do learners with medium level also perform well without help?* The case could be that all types of help require additional effort and lead those learners to aim for a deep-level approach of learning. With the synergy of both helps, they seemed to be able to reach this aim and learning outcomes of approximately 55%. In the no help condition, they had no hint of going deeper or thinking of relations and structures. Thus, learners were free to concentrate on what they were able to understand with their existing knowledge. With this, they also reached approximately 55%. We cannot differentiate what they learned with either both helps or without any help. We can only observe that they reach approximately the same level. Only a differentiated measure of levels of processing such as recall, comprehension, transfer, or structural versus processual tasks could enlighten the quality of the respective learning outcome.

For learners with *high prior knowledge*, they were best supported with a graphic without additional signals or without help. The signals were especially hampering for these learners. Because they were designed to lead learners’ attention to the most relevant concepts on a surface level, they might have interfered with those learners’ approaches to understanding relations on a deeper level. Learners with high prior knowledge were able to distinguish between relevant and irrelevant information and were rather efficient in guiding their attention to what was for them, relevant parts of the learning material. Thus, the signals might have provoked visual attention distribution, have interfered with their usual learning strategies.

The graphic without additional signals helped high knowledgeable learners. It seems to have merged with their existing frame of a mental model without interferences. Again, it would have provided additional insights if we had differentiated the quality of the learning outcomes in terms of different levels of processing and assessed whether those learners reached the deep-level approach with intensive mappings across the given representations, as we assumed.

Summarizing our results, the present findings indicate the importance of considering learners’ prior knowledge, in particular, learners with low prior knowledge seemed to benefit from graphics, signals, or the combination of both supportive elements. Learners with increasing levels of prior knowledge only profited from help, which matches their existing knowledge structures, aims, and strategies. However, as even those learners who would be able to use the help reach only medium levels of learning outcomes, it is plausible that they perceived the given or highlighted information as already known and underestimated the benefit of the additional support (Kalyuga et al., 2003; Kalyuga, 2009). One reason could be that their level of knowledge is still not expertise, and in terms of the literature, they still could be classified as medium knowledgeable learners. It could also be the case that the help we have chosen did not suit their needs for a deep-level approach. Instead, they might have needed different support, for example, prompts, which can foster coherence formation processes (Seufert, 2019) or monitoring (Kauffman et al., 2008).

### Strength and Weaknesses and Recommendations for Further Research

As programming is necessary in many professional fields, overcoming initial barriers to learn to code is of great importance. The present findings outline the importance of considering learners’ prior knowledge for designing learning materials. Our study was integrated into a facultative university course to uncover the beneficial effects of different supporting elements in a real learning setting. This went along with several challenges. Learners had only two weeks to work with the given learning material. Therefore, future studies might investigate the long-term effects of including different instructional means into the learning material for instance during a whole university course to maximize the external validity of the findings. Additionally, to be able to directly compare all experimental conditions, a larger sample size is required. The used experimental design went along with the challenge that the group with instructional means had additional annotations. These emphasized certain content in the learning material as a part of the signaling condition. We ensured that the given informational content of the annotations was also included in all the other conditions as it was displayed in the text. Nevertheless, an additional experimental group without those added annotations integrated could be added in further research to rule out confounding effects.

Motivational aspects were not considered in this study. These might be a considerable factor for explaining the rather low overall learning outcome and should be investigated in further research. Additionally, in this study, learners with high prior knowledge cannot be classified as experts because their knowledge was limited; they reached high levels in comparison to the others. However, reaching expertise in the best sense of the word is a time-consuming and challenging process that takes years.

### Conclusion and Practical Implications

One major challenge is to design adequate learning material for learning abstract content in STEM education, such as learning how to program. Although learners with different prior knowledge can benefit from different supportive elements, these design choices should be made carefully. For *learners with low prior knowledge*, any help is better than none. Therefore, adding UML-charts as well as color coding and mapping lines should be included in the learning material for these learners. *Learners with medium prior knowledge* can be distracted by certain supportive elements with the consequence of a possible decrease in their learning outcome. When including UML-charts or color coding as well as additional lines to map different representations one should carefully consider their benefits and should present additional strategic help to prevent distraction. *Learners with high prior knowledge* seem to also need other types of help to develop their potential. Hence, the learners need more sophisticated supportive elements as adding UML-charts and signals only seems to support to build a basic understanding of the given learning material but did not enable the learners to refine and extend their knowledge. Further research should therefore focus on uncovering the underlying supportive or hampering effects on a more fine-grained level and consider motivational factors.

Furthermore, as aforementioned, different levels of processing in the learning outcome, such as recall, comprehension, and transfer, should be examined. Further research should differentiate between retention, comprehension, and transfer tasks with a sufficient number of items per level. The findings have demonstrated significant differences between levels of processing in dependence when simple recall or more complex transfer tasks were analyzed (e.g., Ozcelik et al., 2010). To further differentiate between these levels of comprehension might lead to further insights into the cognitive learning processes. Such an approach could also be complemented by assessing learners’ aims, that is, their orientation toward superficial or deep learning approaches.

## Data Availability Statement

The raw data supporting the conclusions of this article will be made available by the authors, without undue reservation.

## Ethics Statement

The study was carried out in accordance with the Declaration of Helsinki. Ethical review and approval was not required for the study on human participants in accordance with the local legislation and institutional requirements. The participants provided their written informed consent to participate in this study. Data was used pseudonymously and participants were aware that they had the chance to withdraw their data at any point of the study.

## Author Contributions

TS and IB contributed to the conception and design of the study. IB developed the used material and questionnaires and led data collection for the study. AV analyzed and interpreted the data and drafted the original work. MK wrote the method section. TS and MK critically revised the manuscript. All authors provided approval of the final submitted version of the manuscript and agreed to be accountable for all aspects of the work in ensuring that questions related to the accuracy or integrity of any part of the work are appropriately investigated and resolved.

## Conflict of Interest

The authors declare that the research was conducted in the absence of any commercial or financial relationships that could be construed as a potential conflict of interest.
