# Supplementary material for: Learning From Multiple Representations: Prior Knowledge Moderates the Beneficial Effects of Signals and Abstract Graphics
Source: Front Psychol. 2020 Dec 16;11:601125. doi: 10.3389/fpsyg.2020.601125 (PMC7772191; doi:10.3389/fpsyg.2020.601125)
Supplement: Supplementary file 1 [file Data_Sheet_1.docx]

**Supplemental Material**

**A1. Testing of the model hierarchy of linear and nonlinear regression models**

To test different and multiple linear regression models, different approaches are described in literature. While some approaches are rather data driven other emphasize the importance of theoretical assumption for including the variables and interaction term into the equations (Field, 2013). Although, many researchers chose a rather data driven approach we decided to include the hierarchical regression predictors based on prior empirical studies and theoretical assumptions discussed before. We expect a significant impact of the two different help conditions: graphical help and signals (Clark & Mayer, 2011; Mayer, 2005; Richter, Scheiter, & Eitel, 2016). Furthermore, we expected a significant impact and an interaction between these help conditions with prior knowledge (Kalyuga, 2005). As covariates verbal and spatial ability were included to control for this variance (Höffler & Leutner, 2011; Wallen, Plass, & Brünken, 2005). To compare the different model on the explained variance, namely the *R^2^* of the model can be used as one indicator. As including more variables into this goes along with a higher explained variance per se. To control for this improvement by chance the *adjusted R^2^* is recommended and often used. It compensates for the addition of parameters by only increasing if the new variable enhances the model above what would be obtained by probability. One factor that needs to be considered when calculating multiple regressions is multicollinearity as this might lead to wrong estimated parameters and bias for testing significance. One commonly used indicator for detecting multicollinearity is the *Variance Inflation Factors* (VIF), which should not reach values over 10 (Marquardt, 1970).

All data analyzes were conducted by using R (3.5.1) and Rstudio (1.1.463) using the following additional packages:

library(psych), library(dplyr), library(MVN), library(car), library(ggplot2), library(rockchalk), library(jtools), library(PerformanceAnalytics), library(matrixStats), library(sjPlot), library(sjmisc), library(sjstats), library(parameters).

To test the effect of the different help conditions, signaling and graphical help were included as predictors in the model to analyze their effect on overall learning outcome. To gain further insights in the effects of learners’ aptitudes prior knowledge, spatial and verbal ability were included. Additionally, a quadratic trend for prior knowledge was added based on prior experimental findings and theoretical assumptions. Interaction effects between the named input variables were tested.

*Table 1*. Testing hierarchy of different, nested, multiple regression models including linear and nonlinear trends for prior knowledge and interaction effects

| Multiple Regression | Included Variables |
| --- | --- |
| Main effect model 2 | model 1 + graphical help |
| Main effect model 3 | model 2 + signaling |
| Main effect model 4 | model 3 + prior knowledge^2^ |
| Interaction model 5 | model 4 + prior knowledge^2^ x graphical help |
| Interaction model 6 | model 5 + prior knowledge^2^ x signaling |
| Interaction model 7 | model 6 + graphical help x signaling |
| Interaction model 8 | model 7 + prior knowledge^2^ x graphical help x signaling |

Note: Covariates (spatial and verbal ability) were not included into this overview

*Table 2*. Linear Model 1

| Variable | Estimate | Standard error | *t*-value | *p*-value |
| --- | --- | --- | --- | --- |
| Intercept | -0.31 | 0.59 | -0.52 | .607 |
| Prior knowledge | 1.09 | 0.11 | 10.10 | <.001*** |
| Spatial ability | 6.44 | 2.00 | 3.22 | .002** |
| Verbal ability | 0.38 | 0.12 | 3.14 | .002** |

Note **p*<.05, ***p*<.01, ****p*<.001; *R^2^_adj_* =0.5743

*Table 3.* Linear Model 2

| Variable | Estimate | Standard error | *t*-value | *p*-value |
| --- | --- | --- | --- | --- |
| Intercept | -0.57 | 0.83 | -0.69 | .491 |
| Prior knowledge | 1.09 | 0.11 | 10.07 | <.001*** |
| Graphical help | 0.55 | 1.19 | 0.46 | .646 |
| Spatial ability | 6.37 | 2.01 | 3.16 | .002** |
| Verbal ability | 0.38 | 0.12 | 3.15 | .002** |

Note **p*<.05, ***p*<.01, ****p*<.001; *R^2^*_adj_=0.5715

*Table 4*. Linear Model 3

| Variable | Estimate | Standard error | *t*-value | *p*-value |
| --- | --- | --- | --- | --- |
| Intercept | -0.60 | 1.02 | -0.59 | .557 |
| Prior knowledge | 1.09 | 0.11 | 10.00 | <.001*** |
| Graphical help | 0.55 | 1.19 | 0.46 | .648 |
| Signaling | 0.06 | 1.21 | 0.05 | .961 |
| Spatial ability | 6.35 | 2.05 | 3.11 | .002** |
| Verbal ability | 0.38 | 0.12 | 3.13 | .002** |

Note **p*<.05, ***p*<.01, ****p*<.001; *R^2^*_adj_=0.5678

*Table 5.* Model 4

| Variable | Estimate | Standard error | *t*-value | *p*-value | |
| --- | --- | --- | --- | --- | --- |
| Intercept | -0.92 | 1.11 | -0.83 | | .410 |
| Prior knowledge | 1.11 | 0.11 | 9.89 | | <.001*** |
| Graphical help | 0.53 | 1.20 | 0.44 | | .660 |
| Signaling | 0.05 | 1.21 | 0.04 | | .968 |
| Spatial ability | 6.75 | 2.12 | 3.18 | | .002** |
| Verbal ability | 0.37 | 0.12 | 2.94 | | .004** |
| Prior knowledge^2^ | 0.01 | 0.01 | 0.73 | | .465 |

Note **p*<.05, ***p*<.01, ****p*<.001; *R^2^_adj_*=0.5661

*Table 6.* Model 5

| Variable | Estimate | Standard error | *t*-value | *p*-value |
| --- | --- | --- | --- | --- |
| Intercept | -0.01 | 1.13 | -0.01 | .995 |
| Prior knowledge | 1.14 | 0.11 | 10.41 | <.001*** |
| Graphical help | -1.72 | 1.42 | -1.21 | .227 |
| Signaling | 0.65 | 1.19 | 0.54 | .589 |
| Spatial ability | 6.46 | 2.06 | 3.13 | .002** |
| Verbal ability | 0.39 | 0.12 | 3.17 | .002** |
| Prior knowledge^2^ | -0.03 | 0.02 | -1.48 | .142 |
| Prior knowledge^2^*Graphical help | 0.07 | 0.02 | 2.79 | .006** |

Note **p*<.05, ***p*<.01, ****p*<.001; *R^2^_adj_* =0.5898

*Table 7.* Model 6

| Variable | Estimate | Standard error | *t*-value | *p*-value |
| --- | --- | --- | --- | --- |
| Intercept | -0.43 | 1.22 | -0.35 | .725 |
| Prior knowledge | 1.15 | 0.11 | 10.44 | <.001*** |
| Graphical help | -1.47 | 1.44 | -1.02 | .312 |
| Signaling | 1.49 | 1.50 | 0.99 | .324 |
| Spatial ability | 6.22 | 2.08 | 2.99 | .003** |
| Verbal ability | 0.38 | 0.12 | 3.14 | .002** |
| Prior knowledge^2^ | -0.01 | 0.03 | -0.40 | .690 |
| Prior knowledge^2^*Graphical help | 0.05 | 0.03 | 1.87 | .064 |
| Prior knowledge^2^*Signaling | -0.03 | 0.03 | -0.93 | .357 |

Note **p*<.05, ***p*<.01, ****p*<.001; *R^2^_adj_* =0.5893

*Table 8.* Model 7

| Variable | Estimate | Standard error | *t*-value | *p*-value |
| --- | --- | --- | --- | --- |
| Intercept | 0.15 | 1.32 | 0.11 | .912 |
| Prior knowledge | 1.15 | 0.11 | 10.45 | <.001*** |
| Graphical help | -2.81 | 1.88 | -1.49 | .138 |
| Signaling | 0.19 | 1.90 | 0.10 | .920 |
| Spatial ability | 5.93 | 2.09 | 2.83 | .005** |
| Verbal ability | 0.39 | 0.12 | 3.22 | .002** |
| Prior knowledge^2^ | -0.01 | 0.03 | -0.39 | .701 |
| Prior knowledge^2^*Graphical help | 0.05 | 0.03 | 1.91 | .059 |
| Prior knowledge^2^*Signaling | -0.03 | 0.03 | -0.87 | .387 |
| Graphical help*Signaling | 2.64 | 2.38 | 1.11 | .269 |

Note **p*<.05, ***p*<.01, ****p*<.001; *R^2^_adj_* =0.5901

*Table 9.* Model 8

| Variable | Estimate | Standard error | *t*-value | *p*-value |
| --- | --- | --- | --- | --- |
| Intercept | 0.80 | 1.34 | 0.60 | .551 |
| Prior knowledge | 1.17 | 0.11 | 10.74 | <.001*** |
| Graphical help | -3.90 | 1.93 | -2.02 | .046* |
| Signaling | -1.11 | 1.98 | -0.56 | .576 |
| Spatial ability | 5.46 | 2.08 | 2.63 | .010* |
| Verbal ability | 0.40 | 0.12 | 3.34 | .001** |
| Prior knowledge^2^ | -0.04 | 0.03 | -1.26 | .210 |
| Graphical help*Signaling | 6.41 | 2.98 | 2.15 | .034* |
| Prior knowledge^2^ *Graphical help | 0.09 | 0.03 | 2.72 | .008** |
| Prior knowledge^2^ *Signaling | 0.02 | 0.04 | 0.43 | .670 |
| Prior knowledge^2^ *Graphical help*Signaling | -0.13 | 0.06 | -2.05 | .043* |

Note **p*<.05, ***p*<.01, ****p*<.001; *R^2^* =0.6013; *VIF*=6.10

Comparing the different regression models based on the R^2^_adj_ the best model which explains most variance is quadratic model 8 (model 7 & model 8: *F*(1,113)=4.19, *p*=.043; see *Table 9*).

In the following additional data for analyzing the chose model in regard to potential outliers and assumptions are added. To analyze homoscedasticity we displayed the distribution of residuals which should be randomly and evenly dispersed throughout the plot. This assumption seems supported by the current data (see figure 1).


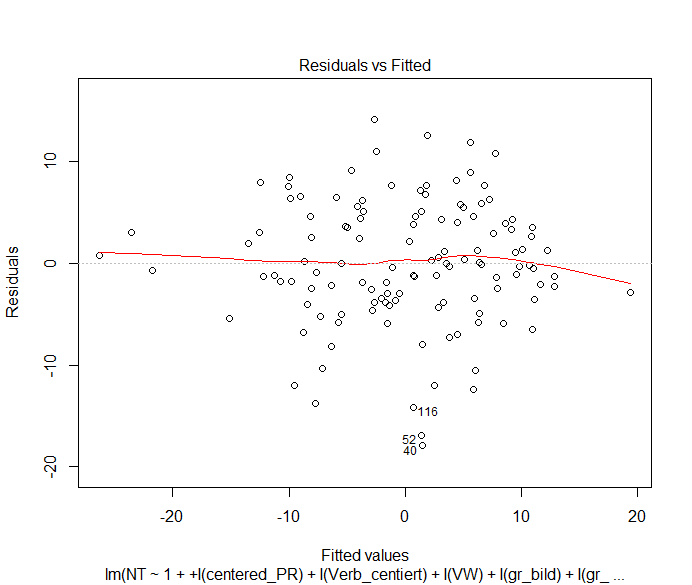


*Figure 1.* Residuals and fitted values of the chosen quadratic model 8


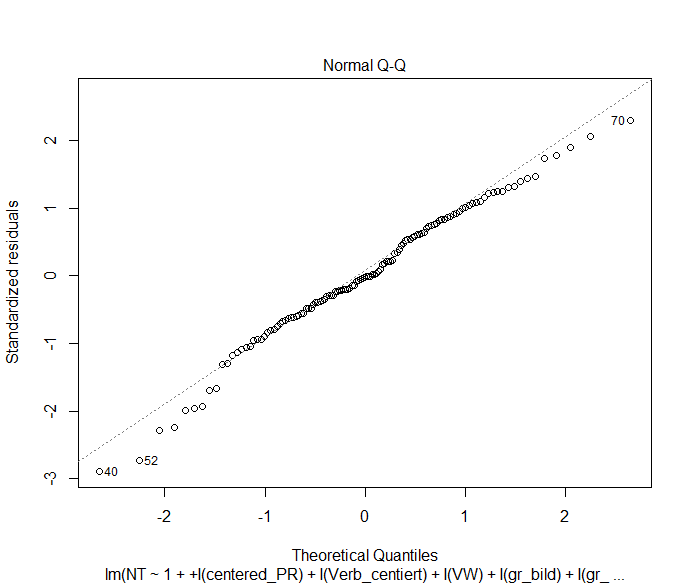


*Figure 2.* QQ-plot of the residuals of the chosen quadratic model 8

In figure 2 the QQ-plot is displayed to detect deviations from the normal distribution. Only extreme values +/- 2 standard deviations substantial deviations from the optimal distribution was detected. Therefore, the distribution of the residuals based on visual inspection of the QQ-plot is sufficiently normal.


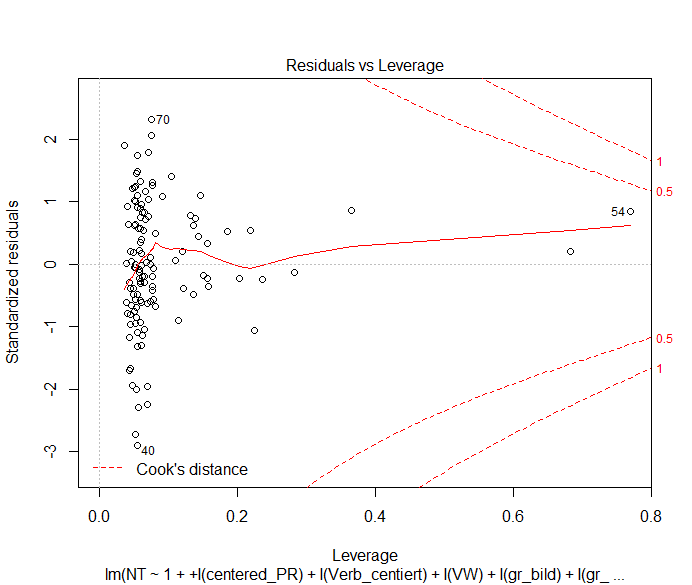
Based on Cook’s distance no additional outliers were detected as none of the values was higher than 1 (see figure 3; Field, 2013).

*Figure 3.* Cook’s distance graph of the chosen quadratic model 8

**A2. Learning Material**

The learning material was a booklet with a maximum of 18 pages. Depending on the experimental group, the learning material contained additional information about the supporting elements (graphics or signals). The booklet started with a title page (page 1) and a page with an introduction to the topic and its relevance (page 2). The third page provided information about characteristics of the learning material and should help the students with the signals and/or UML-diagrams (page 3). This page was only included in the booklet of the experimental groups 2 (with graphics), 3 (with signals), and 4 (with signals and graphics), and was modified according to each of the experimental group’s conditions. The next pages (page 4-9) were dealing with the topic “classes and objects”. An additional page (page 10) included three tasks to exercise and to test oneself. Furthermore, how students rated the complexity of the content, their mental load during learning and fun while dealing with the topic has been assessed (page 11). The second topic of the learning material was *linear lists* and was presented on five pages (page 12-16). For this topic, a self-test with two questions was included (page 17) and again the complexity of the content, mental load during learning and fun while dealing with the topic have been assessed (page 18).

**A3. References**

Atkinson, A. C. (1981). Likelihood ratios, posterior odds and information criteria. *Journal of Econometrics*, *16*(1), 15–20. https://doi.org/10.1016/0304-4076(81)90072-5

Clark, R. C., & Mayer, R. E. (2011). *E-Learning and the science of instruction: Proven guidelines for consumers and designers of multimedia learning* (3. ed.). *Pfeiffer essential resources for training and HR professionals*. San Francisco, Calif.: Pfeiffer.

Field, A. (2013). *Discovering statistics using IBM SPSS statistics: And sex and drugs and rock 'n' roll* (4th edition). *MobileStudy*. Los Angeles, London, New Delhi, Singapore, Washington DC: SAGE.

Höffler, T. N., & Leutner, D. (2011). The role of spatial ability in learning from instructional animations – Evidence for an ability-as-compensator hypothesis. *Computers in Human Behavior*, *27*(1), 209–216. https://doi.org/10.1016/j.chb.2010.07.042

Kalyuga, S. (2005). Prior Knowlege Principle in Multimedia Learning. In R. E. Mayer (Ed.), *The Cambridge handbook of multimedia learning* (pp. 325–338). Cambridge, New York: Cambridge University Press.

Kuha, J. (2004). AIC and BIC. *Sociological Methods & Research*, *33*(2), 188–229. https://doi.org/10.1177/0049124103262065

Mayer, R. E. (2005). Principles for Reducing Extraneous Processing in Multimedia Learning: Coherence, Signaling, Redundancy, Spatial Contiguity, and Temporal Contiguity Principles. In R. E. Mayer (Ed.), *The Cambridge handbook of multimedia learning* (pp. 183–200). Cambridge, New York: Cambridge University Press.

Richter, J., Scheiter, K., & Eitel, A. (2016). Signaling text-picture relations in multimedia learning: A comprehensive meta-analysis. *Educational Research Review*, *17*, 19–36. https://doi.org/10.1016/j.edurev.2015.12.003

Vrieze, S. I. (2012). Model selection and psychological theory: A discussion of the differences between the Akaike information criterion (AIC) and the Bayesian information criterion (BIC). *Psychological Methods*, *17*(2), 228–243. https://doi.org/10.1037/a0027127

Wallen, E., Plass, J. L., & Brünken, R. (2005). The function of annotations in the comprehension of scientific texts: Cognitive load effects and the impact of verbal ability. *Educational Technology Research and Development*, *53*(3), 59–71. https://doi.org/10.1007/BF02504798
